# The pentacyclic triterpenoid phytosterol lupeol promotes antioxidant response in the nematode *C. elegans*

**DOI:** 10.17912/micropub.biology.000581

**Published:** 2022-06-01

**Authors:** Anna Voia, Vincent Poupart, Jean-Claude Labbé

**Affiliations:** 1 Collège Jean-de-Brébeuf, 3200, chemin de la Côte-Sainte-Catherine, Montréal (Québec) H3T 1C1, Canada; 2 Institute for Research in Immunology and Cancer (IRIC), Université de Montréal, C.P. 6128, Succ. Centre-ville, Montréal, QC, H3C 3J7, Canada; 3 Department of Pathology and Cell Biology, Université de Montréal, C.P. 6128, Succ. Centre-ville, Montréal, QC, H3C 3J7, Canada

## Abstract

Plants of the Mimosa genus are studied and used for their bioactive properties. Among bioactive phytochemicals are quercetin and myricetin, which have been demonstrated to act as antioxidants in many contexts (Taheri et al. 2020; Xu et al. 2019), including in *C. elegans* (Buchter et al. 2013; Grünz et al. 2012; Sugawara and Sakamoto 2020). Other phytochemicals from these plants, such as the triterpenoid phytosterol lupeol, have been shown to have antioxidant properties but have not been as extensively characterized in model organisms (Liu et al. 2021; Shai et al. 2009). Here we employed the nematode *C. elegans* to assess whether lupeol elicits antioxidant response *in vivo* . Using reporter assays for oxidative stress, we find that treatment of animals with lupeol rescues some of the effects resulting from treatment with the prooxidant paraquat. Our results demonstrate that lupeol displays antioxidant properties *in vivo* in *C. elegans* .

**Figure 1. Monitoring of oxidative response in live C. elegans animals.**
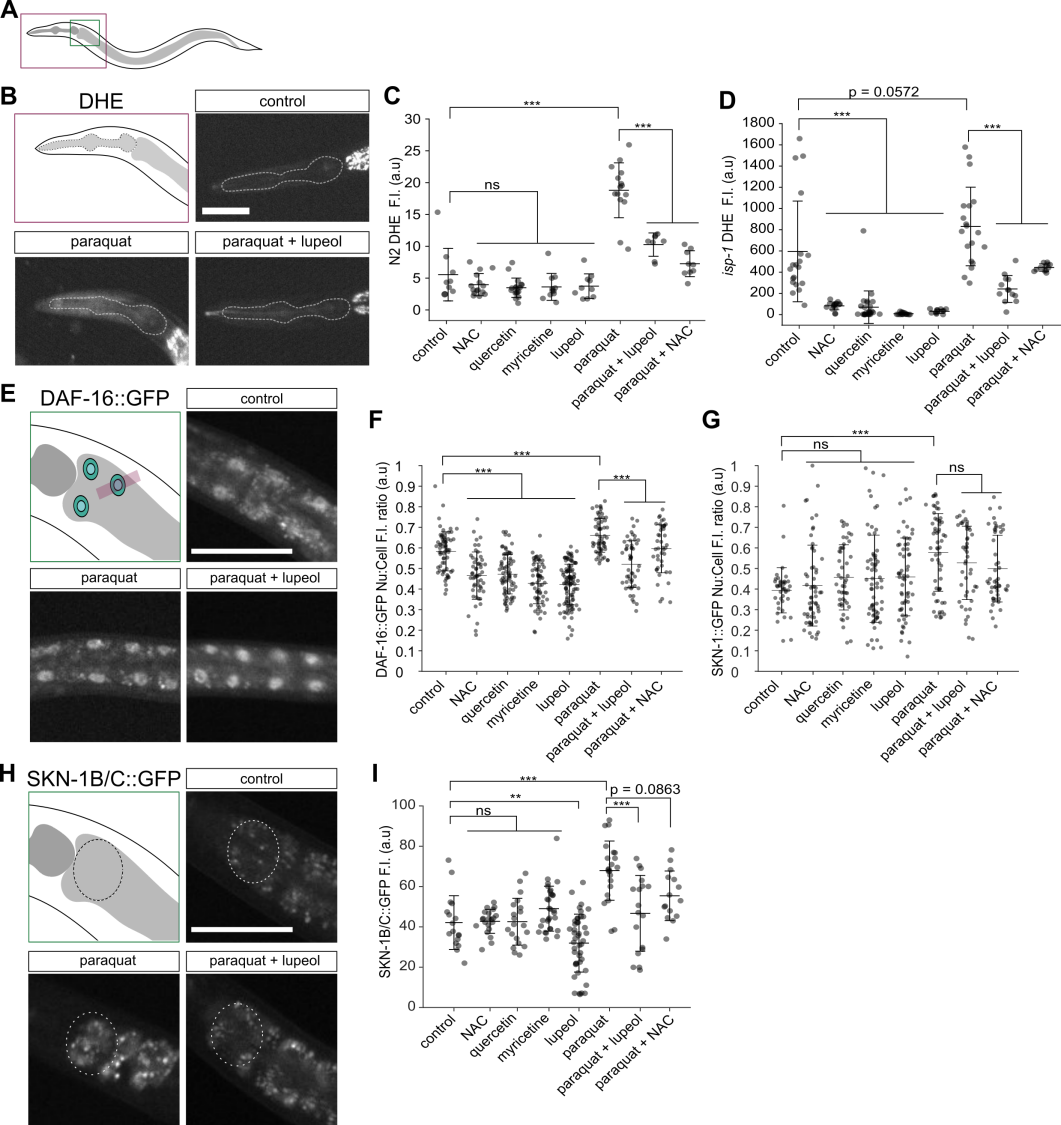
**(A)** Schematic representation of *C. elegans* showing the regions of the animals that were imaged. **(B)** Epifluorescence images of wild-type (N2) L4 larvae treated with dihydroethidium. **(C-D)** Fluorescence levels of dihydroethidium measured in the pharynx of wild-type animals (N2; C) and *isp-1(qm150)* mutants (D) grown in presence of the compounds indicated on the horizontal axis. **(E)** Epifluorescence images of adult animals expressing DAF-16::GFP. **(F-G)** Nuclear:total cell (Nu:Cell) fluorescence ratio of DAF-16::GFP (F) and SKN-1B/C::GFP (G) measured in intestinal cells of adult animals grown in presence of the compounds indicated on the horizontal axis. **(H)** Epifluorescence images of adult animals expressing SKN-1B/C::GFP. **(I)** Total fluorescence levels of SKN-1B/C::GFP measured in the intestine of adult animals grown in presence of the compounds indicated on the horizontal axis. In panels B, E and H, the schematic representation at the top left outlines the region where fluorescence levels were measured, and the images are taken from animals grown in control (top right), paraquat (bottom left) and paraquat+lupeol (bottom right) conditions. For all images, scale bar = 50 µm. For all graphs, black lines represent mean ± standard deviation, and statistical analyses were done using a one-way ANOVA test with a Tukey-Kramer *post hoc* test (*** = p < 0.001, ** = p < 0.01, ns = p > 0.05). F.I. = fluorescence intensity, a.u. = arbitrary units.

## Description

To assess the antioxidant properties of lupeol, we first employed the fluorescent, ROS-sensitive dye dihydroethidium (DHE) to measure changes in the levels of ROS *in vivo* (Senchuk et al. 2018). This was done in either wild-type animals (N2) or *isp-1(qm150)* mutants, which are defective in complex III of the mitochondrial electron transport chain and thus intrinsically have elevated levels of ROS (Yang and Hekimi 2010). Accordingly, the average fluorescence level of DHE measured in the pharynx of *isp-1* mutants (595.7±474.7) was higher than that measured in wild-type animals (5.6±4.1; p=0.001, unpaired Student's *t* -test). Exposing either strain to the prooxidant paraquat resulted in a significant increase of DHE fluorescence, consistent with elevation of ROS levels (Figure 1A-D). In both strains we found that lupeol impacted DHE fluorescence levels in a manner similar to that of the previously-characterized antioxidants N-acetyl-L-cysteine (NAC), quercetin and myricetin: it had no measurable effect in wild-type animals but significantly reduced DHE fluorescence in *isp-1(qm150)* mutants (Figure 1C-D). This suggested that lupeol lowers ROS levels in conditions where they are elevated. To further assess this, we asked whether exposing animals to lupeol could mitigate the effect of paraquat on ROS levels. We found that the increase of DHE fluorescence resulting from exposure to paraquat was rescued by co-treatment with lupeol in both wild-type and *isp-1(qm150)* animals, an effect also obtained when co-treating animals with the antioxidant NAC (Figure 1C-D). These results suggest that the treatment of *C. elegans* with lupeol can decrease ROS levels *in vivo* .

To confirm these results and further explore the pathways that are impacted by lupeol, we monitored the activity of two transcription factors known to mediate response to oxidative stress *in vivo* , the FOXO homolog DAF-16 and the Nrf2 homolog SKN-1 (Ayuda-Duran et al. 2020; Blackwell et al. 2015; Hekimi et al. 2016). This was done using strains expressing these transcription factors fused to a fluorescent protein (Aghayeva et al. 2020; An and Blackwell 2003), thus enabling the monitoring of their localization and levels in *C. elegans* intestinal cells (Figure E, H). As reported previously (An and Blackwell 2003; Henderson and Johnson 2001), treating animals with paraquat caused an increase in the fraction of both DAF-16 and SKN-1B/C found in the nucleus of intestinal cells compared to the total cellular levels (Figure 1E-G). The changes in nuclear:total cell ratio of DAF-16 due to paraquat treatment were rescued by co-treating animals with lupeol or NAC (Figure 1F). Treatment with lupeol or NAC had no impact on the nuclear:total cell ratio of SKN-1B/C following paraquat treatment (Figure 1G). However, the total fluorescence levels of SKN-1B/C::GFP in the intestine of adult animals revealed that they increase after treatment with paraquat and that this increase is rescued by co-treatment with lupeol (Figure 1H-I). These results indicate that, like known antioxidants, lupeol can mitigate some of the response to oxidative stress in *C. elegans* intestinal cells.

Together, our results support the notion that lupeol is an antioxidant compound that impacts ROS levels and decreases oxidative stress *in vivo* in *C. elegans* . They further support findings made in other systems demonstrating lupeol's antioxidant properties (Liu et al. 2021; Shai et al. 2009).

## Methods

***C. elegans* strain maintenance**

Animals were grown on NGM plates seeded with *E. coli* strain OP50 and maintained at either 15ºC (N2, MQ887) or 20ºC (MQ1844, OH16024) as described (Brenner 1974). Prior to assays with compounds, worms were synchronized as first stage (L1) larvae by dissolving gravid hermaphrodites in sodium hypochlorite solution (1.2% NaOCl, 250 mM KOH) and hatching recovered embryos for 24h at 15ºC in M9 buffer (22.04 mM KH _2_ PO _4_ , 42.27 mM Na _2_ HPO _4_ , 85.55 mM NaCl, 1 mM MgSO _4_ ).

**Treatments with chemical compounds**

Chemical compounds were acquired from Sigma and solubilized in water (paraquat, N-acetyl-L-cysteine [NAC]) or dimethyl sulfoxide (DMSO; quercetin, myricetin, lupeol). Each compound was spread onto the surface of NGM plates to the following final concentrations: paraquat (300 µM), NAC (5 mM), quercetin (100 µM), myricetin (100 µM) and lupeol (100 µM); note that lupeol is poorly soluble in water and therefore the final concentration was likely lower. All plates including control contained DMSO at a final concentration of 1% (v/v). After brief drying, plates were seeded with *E. coli* OP50 and left for 24h at room temperature to allow bacterial growth (and compound diffusion into the NGM medium). Synchronized L1-stage animals (15-20 individuals) were added to each plate and incubated at 15ºC or 20ºC for 2-3 days, until they had reached the L4 or adult stage. Animals were then collected in M9 buffer prior to further processing.

**Dihydroethidium (DHE) treatment**

L4-stage animals were resuspended in M9 buffer supplemented with DHE (Sigma) at a final concentration of 30 µM (from a 30 mM stock solubilized in DMSO) and incubated with mild agitation for 1h at room temperature. Animals were washed thrice with M9 buffer before mounting and imaging.

**Imaging and image analysis**

Animals were immobilized in M9 buffer supplemented with 0.04% tetramisole and mounted on a 3% agarose pad. A coverslip was applied and corners were sealed with VaLaP (1:1:1 Vaseline, lanolin, and paraffin). Images were acquired with a Zeiss HRM camera (Carl Zeiss Canada Ltd., Toronto, Canada) mounted on a Zeiss Axio-Imager Z1 microscope, using a Plan Apochromat 20x/0.8 NA objective. Mid-plane sections of the mounted samples were acquired using both DIC optics and 488 nm epifluorescence illumination, and the acquisition system was controlled by Axiovision software. Fluorescence levels were measured using ImageJ software (NIH).

DHE fluorescence intensity was measured in the pharynx of each animal. Average raw integrated fluorescence density was obtained for a region of interest (ROI) encompassing the entire pharynx within an imaging plane. Average background measured outside of the animal for an ROI of the same size was subtracted.

The fluorescence intensity and distribution of transgenic reporters was measured in the animals' intestine. For levels of SKN-1B/C::GFP fluorescence, average raw integrated density was measured in a circular ROI defined in the foregut region of each animal, just below (posterior of) the pharynx, and average background measured outside of the animal was subtracted. For measurements of the nuclear:total cell ratio of DAF-16::GFP and SKN-1B/C::GFP, fluorescence intensity was measured along a 10 pixel-wide line drawn across a cell, and the peak value of fluorescence intensity measured in the nuclear region was divided by the average fluorescence intensity measured in the entire cell, both after background subtraction.

**Graphing and statistical analysis**

Results were graphed using MATLAB (The MathWorks Inc, R2020b v9.9.0.1592791). Statistical p values were determined after performing ANOVA with Tukey-Kramer post-hoc analysis.

## Reagents

**Table:** 

**Strain**	**Genotype**	**Available from**
N2	*Wild type*	CGC
MQ887	*isp-1(qm150) IV*	CGC
MQ1844	*ldIs7[skn-1b/c::GFP + rol-6(su1006)]*	CGC
OH16024	*daf-16(ot971[daf-16::GFP]) I*	CGC
